# Poly[μ_2_-aqua-aqua-μ_5_-naphthalene-2,7-disulfonato-strontium]

**DOI:** 10.1107/S1600536811047313

**Published:** 2011-11-19

**Authors:** Shan Gao, Seik Weng Ng

**Affiliations:** a Key Laboratory of Functional Inorganic Material Chemistry, Ministry of Education, Heilongjiang University, Harbin 150080, People’s Republic of China; b Department of Chemistry, University of Malaya, 50603 Kuala Lumpur, Malaysia; c Chemistry Department, Faculty of Science, King Abdulaziz University, PO Box 80203 Jeddah, Saudi Arabia

## Abstract

In the crystal structure of the polymeric title compound, [Sr(C_10_H_6_O_6_S_2_)(H_2_O)_2_]_*n*_, the naphthalene-2,7-disulfonate dianion uses one –SO_3_ unit to bind to two Sr^II^ cations and the other –SO_3_ unit to bind to three Sr^II^ cations; of the two coordinated water mol­ecules, one is monodentate to one Sr^II^ cation, whereas the other bridges two Sr^II^ cations. The μ_5_-bridging mode of the dianon and the μ_2_-bridging mode of the water mol­ecule generate a polymeric three-dimensional network which is consolidated by O—H⋯O hydrogen bonds. The Sr^II^ cation exists in an undefined eight-coordinate environment.

## Related literature

For a review of metal arene­sulfonates, see: Cai (2004 ▶). For a related strontium naphthalene­disulfonate, see: Cai *et al.* (2001 ▶). 
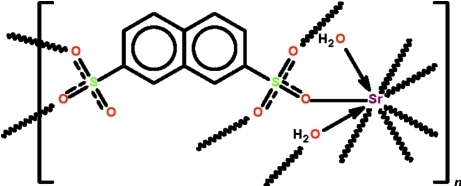


## Experimental

### 

#### Crystal data

[Sr(C_10_H_6_O_6_S_2_)(H_2_O)_2_]
*M* *_r_* = 409.92
Orthorhombic, 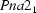

*a* = 13.064 (6) Å
*b* = 19.324 (9) Å
*c* = 5.1989 (17) Å
*V* = 1312.5 (9) Å^3^
*Z* = 4
Mo *K*α radiation
μ = 4.46 mm^−1^
*T* = 293 K
0.18 × 0.12 × 0.12 mm

#### Data collection

Rigaku R-AXIS RAPID IP diffractometer
Absorption correction: multi-scan (*ABSCOR*; Higashi, 1995 ▶) *T*_min_ = 0.501, *T*_max_ = 0.616
11845 measured reflections
2962 independent reflections
2646 reflections with *I* > 2σ(*I*)
*R*_int_ = 0.040

#### Refinement

*R*[*F* ^2^ > 2σ(*F* ^2^)] = 0.028
*wR*(*F* ^2^) = 0.071
*S* = 1.04
2962 reflections
190 parameters
1 restraint
H-atom parameters constrained
Δρ_max_ = 0.60 e Å^−3^
Δρ_min_ = −0.51 e Å^−3^
Absolute structure: Flack (1983 ▶), 1584 Friedel pairs
Flack parameter: −0.011 (6)

### 

Data collection: *RAPID-AUTO* (Rigaku, 1998 ▶); cell refinement: *RAPID-AUTO*; data reduction: *CrystalClear* (Rigaku/MSC, 2002 ▶); program(s) used to solve structure: *SHELXS97* (Sheldrick, 2008 ▶); program(s) used to refine structure: *SHELXL97* (Sheldrick, 2008 ▶); molecular graphics: *X-SEED* (Barbour, 2001 ▶); software used to prepare material for publication: *publCIF* (Westrip, 2010 ▶).

## Supplementary Material

Crystal structure: contains datablock(s) global, I. DOI: 10.1107/S1600536811047313/xu5381sup1.cif

Structure factors: contains datablock(s) I. DOI: 10.1107/S1600536811047313/xu5381Isup2.hkl

Additional supplementary materials:  crystallographic information; 3D view; checkCIF report

## Figures and Tables

**Table 1:** Selected bond lengths (Å)

Sr1—O1	2.612 (2)
Sr1—O2^i^	2.494 (2)
Sr1—O3^ii^	2.595 (2)
Sr1—O5^iii^	2.549 (2)
Sr1—O6^iv^	2.540 (2)
Sr1—O1*w*	2.614 (2)
Sr1—O2*w*	2.756 (3)
Sr1—O2*w*^v^	2.974 (3)

Symmetry codes: (i) 
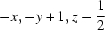
; (ii) 
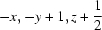
; (iii) 
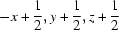
; (iv) 
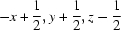
; (v) 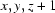.

**Table 2:** Hydrogen-bond geometry (Å, °)

*D*—H⋯*A*	*D*—H	H⋯*A*	*D*⋯*A*	*D*—H⋯*A*
O1*w*—H1*w*1⋯O4^vi^	0.84	2.29	3.066 (4)	154
O1*w*—H1*w*2⋯O4^vii^	0.84	2.27	2.904 (4)	132
O2*w*—H2*w*2⋯O4^vii^	0.84	2.03	2.856 (3)	167

Symmetry codes: (vi) 
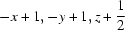
; (vii) 
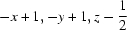
.

**Fig. 1.**
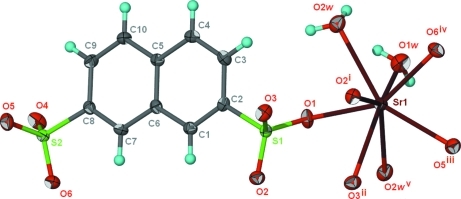
Thermal ellipsoid plot (Barbour, 2001) of a fragment of polymeric Sr(H2O)2(C10H6O6S2) at the 50% probability level; hydrogen atoms are drawn as spheres of arbitrary radius.

## supplementary crystallographic information

### Comment

A review of metal arenesulfonates that are synthesized in aqueous medium explains the reasons for the ability of the ions to form stable metal-organic frameworks owing to multiple coordination modes of the sulfonate –SO_3_ groups (Cai, 2004). Among the divalent metal derivatives, the strontium system has been less studied (Cai *et al.*, 2001). In the crystal structure of Sr(H_2_O)_2_(C_10_H_6_O_6_S_2_), the C_10_H_6_O_6_S_2_^2-^ dianion uses one –SO_3_ unit to bind to two Sr^II^ atoms and the other –SO_3_ unit to bind to three Sr^II^ atoms; of the two water molecules, one is monodentate to one Sr atom whereas the other bridge two Sr atoms (Scheme I, Fig. 1). The *µ*_5_-bridging mode of the dianon and the *µ*_2_-bridging mode of the water molecule generates a polymeric three-dimensional network; the network is consolidated by O–H···O hydrogen bonds (Table 1). The Sr atom exists in an undefined eight-coordinate environment.

### Experimental

Strontium nitrate (1 mmol) and sodium naphthalene-2,7-disulfonate (1 mmol) were dissolved in water (10 ml). The solution was filtered and set aside; colorless crystals were isolated from the filtrate after several days.

### Refinement

Hydrogen atoms were generated geometrically and were included in the riding model approximation [C—H 0.93 Å and O–H 0.84 Å, *U* 1.2 to 1.5*U*_eq_(C,O)]. The 3 7 2 reflection was omitted owing to bad agreement.

### Crystal data

**Table:**

[Sr(C_10_H_6_O_6_S_2_)(H_2_O)_2_]	*F*(000) = 816
*M**_r_* = 409.92	*D*_x_ = 2.075 Mg m^−^^3^
Orthorhombic, *P**n**a*2_1_	Mo *K*α radiation, λ = 0.71073 Å
Hall symbol: P 2c -2n	Cell parameters from 10271 reflections
*a* = 13.064 (6) Å	θ = 3.1–27.5°
*b* = 19.324 (9) Å	µ = 4.46 mm^−^^1^
*c* = 5.1989 (17) Å	*T* = 293 K
*V* = 1312.5 (9) Å^3^	Prism, colorless
*Z* = 4	0.18 × 0.12 × 0.12 mm

### Data collection

**Table:**

Rigaku R-AXIS RAPID IP diffractometer	2962 independent reflections
Radiation source: fine-focus sealed tube	2646 reflections with *I* > 2σ(*I*)
graphite	*R*_int_ = 0.040
ω scan	θ_max_ = 27.5°, θ_min_ = 3.1°
Absorption correction: multi-scan (*ABSCOR*; Higashi, 1995)	*h* = −16→16
*T*_min_ = 0.501, *T*_max_ = 0.616	*k* = −24→25
11845 measured reflections	*l* = −6→6

### Refinement

**Table:**

Refinement on *F*^2^	Secondary atom site location: difference Fourier map
Least-squares matrix: full	Hydrogen site location: inferred from neighbouring sites
*R*[*F*^2^ > 2σ(*F*^2^)] = 0.028	H-atom parameters constrained
*wR*(*F*^2^) = 0.071	*w* = 1/[σ^2^(*F*_o_^2^) + (0.0383*P*)^2^] where *P* = (*F*_o_^2^ + 2*F*_c_^2^)/3
*S* = 1.04	(Δ/σ)_max_ = 0.001
2962 reflections	Δρ_max_ = 0.60 e Å^−^^3^
190 parameters	Δρ_min_ = −0.51 e Å^−^^3^
1 restraint	Absolute structure: Flack (1983), 1584 Friedel pairs
Primary atom site location: structure-invariant direct methods	Flack parameter: −0.011 (6)

### Fractional atomic coordinates and isotropic or equivalent isotropic displacement parameters (Å^2^)

**Table:**

	*x*	*y*	*z*	*U*_iso_*/*U*_eq_	
Sr1	−0.008465 (18)	0.635847 (12)	0.49937 (9)	0.02043 (9)	
S1	0.14037 (4)	0.47764 (3)	0.49983 (18)	0.01857 (14)	
S2	0.64351 (5)	0.24848 (3)	0.49376 (18)	0.01863 (14)	
O1	0.14590 (15)	0.55148 (11)	0.5589 (4)	0.0294 (6)	
O2	0.12497 (16)	0.43544 (11)	0.7268 (4)	0.0256 (5)	
O3	0.06622 (16)	0.46443 (12)	0.2980 (4)	0.0285 (5)	
O4	0.74894 (15)	0.27350 (11)	0.5219 (6)	0.0310 (5)	
O5	0.63559 (18)	0.19599 (11)	0.2968 (4)	0.0294 (5)	
O6	0.59963 (17)	0.22808 (12)	0.7371 (4)	0.0288 (5)	
O1w	0.13881 (15)	0.72744 (11)	0.5039 (7)	0.0376 (5)	
H1w1	0.1543	0.7369	0.6567	0.056*	
H1w2	0.1903	0.7113	0.4278	0.056*	
O2w	0.08346 (17)	0.63163 (10)	0.0225 (6)	0.0315 (5)	
H2w1	0.1187	0.5953	0.0242	0.038*	
H2w2	0.1257	0.6644	0.0242	0.038*	
C1	0.3192 (2)	0.40460 (13)	0.4846 (8)	0.0208 (5)	
H1	0.2958	0.3826	0.6325	0.025*	
C2	0.2615 (2)	0.45518 (15)	0.3713 (5)	0.0187 (6)	
C3	0.2939 (2)	0.48863 (15)	0.1474 (6)	0.0240 (7)	
H3	0.2526	0.5222	0.0717	0.029*	
C4	0.3857 (2)	0.47221 (15)	0.0399 (6)	0.0230 (7)	
H4	0.4072	0.4949	−0.1083	0.028*	
C5	0.4485 (2)	0.42073 (15)	0.1525 (6)	0.0195 (6)	
C6	0.4143 (2)	0.38578 (15)	0.3764 (6)	0.0196 (6)	
C7	0.4773 (2)	0.33370 (14)	0.4836 (9)	0.0205 (6)	
H7	0.4555	0.3097	0.6287	0.025*	
C8	0.5694 (2)	0.31837 (15)	0.3767 (5)	0.0196 (6)	
C9	0.6062 (3)	0.35484 (15)	0.1587 (6)	0.0247 (7)	
H9	0.6707	0.3451	0.0920	0.030*	
C10	0.5462 (2)	0.40419 (15)	0.0481 (6)	0.0241 (7)	
H10	0.5694	0.4274	−0.0974	0.029*	

### Atomic displacement parameters (Å^2^)

**Table:**

	*U*^11^	*U*^22^	*U*^33^	*U*^12^	*U*^13^	*U*^23^
Sr1	0.02020 (14)	0.02060 (13)	0.02050 (13)	−0.00055 (9)	−0.00006 (17)	0.00153 (17)
S1	0.0168 (3)	0.0174 (3)	0.0215 (3)	0.0006 (2)	0.0002 (4)	−0.0011 (4)
S2	0.0182 (3)	0.0182 (3)	0.0195 (3)	−0.0003 (2)	−0.0002 (4)	0.0013 (4)
O1	0.0256 (12)	0.0186 (10)	0.0439 (18)	0.0013 (8)	0.0031 (10)	−0.0054 (9)
O2	0.0238 (12)	0.0276 (12)	0.0252 (11)	−0.0004 (9)	0.0028 (9)	0.0027 (9)
O3	0.0219 (12)	0.0363 (13)	0.0273 (11)	0.0012 (10)	−0.0039 (10)	−0.0047 (10)
O4	0.0206 (10)	0.0311 (11)	0.0414 (13)	−0.0040 (8)	−0.0073 (12)	0.0085 (14)
O5	0.0360 (14)	0.0235 (12)	0.0287 (12)	0.0056 (9)	−0.0051 (11)	−0.0047 (10)
O6	0.0355 (14)	0.0249 (11)	0.0260 (12)	0.0005 (10)	0.0050 (10)	0.0045 (10)
O1w	0.0341 (12)	0.0421 (13)	0.0366 (12)	−0.0059 (9)	−0.0008 (16)	−0.0050 (16)
O2w	0.0313 (11)	0.0307 (11)	0.0324 (13)	−0.0031 (9)	0.0037 (14)	−0.0060 (11)
C1	0.0234 (13)	0.0192 (12)	0.0197 (12)	−0.0033 (10)	−0.0004 (17)	−0.0003 (15)
C2	0.0143 (14)	0.0210 (14)	0.0210 (14)	−0.0008 (11)	−0.0003 (11)	−0.0046 (12)
C3	0.0243 (17)	0.0208 (14)	0.0270 (15)	0.0016 (12)	−0.0040 (14)	0.0028 (13)
C4	0.0253 (15)	0.0245 (14)	0.0193 (19)	−0.0021 (11)	−0.0002 (13)	0.0025 (12)
C5	0.0208 (16)	0.0184 (14)	0.0192 (15)	−0.0036 (12)	−0.0012 (12)	−0.0001 (12)
C6	0.0210 (16)	0.0159 (14)	0.0219 (14)	−0.0016 (12)	−0.0027 (12)	−0.0023 (12)
C7	0.0221 (13)	0.0210 (13)	0.0185 (14)	−0.0015 (10)	−0.0001 (16)	0.0049 (18)
C8	0.0224 (16)	0.0162 (14)	0.0202 (13)	0.0005 (12)	−0.0027 (12)	−0.0015 (11)
C9	0.0236 (17)	0.0257 (16)	0.0248 (16)	−0.0001 (12)	0.0064 (14)	0.0002 (13)
C10	0.0271 (16)	0.0243 (15)	0.021 (2)	0.0001 (12)	0.0041 (12)	0.0032 (12)

### Geometric parameters (Å, °)

**Table:**

Sr1—O1	2.612 (2)	O1w—H1w2	0.8400
Sr1—O2^i^	2.494 (2)	O2w—Sr1^viii^	2.974 (3)
Sr1—O3^ii^	2.595 (2)	O2w—H2w1	0.8400
Sr1—O5^iii^	2.549 (2)	O2w—H2w2	0.8400
Sr1—O6^iv^	2.540 (2)	C1—C2	1.367 (4)
Sr1—O1w	2.614 (2)	C1—C6	1.412 (4)
Sr1—O2w	2.756 (3)	C1—H1	0.9300
Sr1—O2w^v^	2.974 (3)	C2—C3	1.397 (4)
S1—O2	1.448 (2)	C3—C4	1.360 (4)
S1—O3	1.451 (2)	C3—H3	0.9300
S1—O1	1.461 (2)	C4—C5	1.417 (4)
S1—C2	1.772 (3)	C4—H4	0.9300
S2—O6	1.444 (2)	C5—C6	1.418 (4)
S2—O5	1.445 (2)	C5—C10	1.423 (4)
S2—O4	1.467 (2)	C6—C7	1.414 (4)
S2—C8	1.769 (3)	C7—C8	1.358 (4)
O2—Sr1^ii^	2.494 (2)	C7—H7	0.9300
O3—Sr1^i^	2.595 (2)	C8—C9	1.418 (4)
O5—Sr1^vi^	2.549 (2)	C9—C10	1.362 (4)
O6—Sr1^vii^	2.540 (2)	C9—H9	0.9300
O1w—H1w1	0.8400	C10—H10	0.9300
			
O2^i^—Sr1—O6^iv^	78.25 (8)	S1—O3—Sr1^i^	139.31 (13)
O2^i^—Sr1—O5^iii^	101.47 (8)	S2—O5—Sr1^vi^	142.79 (14)
O6^iv^—Sr1—O5^iii^	72.56 (8)	S2—O6—Sr1^vii^	147.99 (15)
O2^i^—Sr1—O3^ii^	75.53 (8)	Sr1—O1w—H1w1	109.5
O6^iv^—Sr1—O3^ii^	135.12 (7)	Sr1—O1w—H1w2	109.5
O5^iii^—Sr1—O3^ii^	77.77 (8)	H1w1—O1w—H1w2	109.5
O2^i^—Sr1—O1	101.17 (7)	Sr1—O2w—Sr1^viii^	130.23 (8)
O6^iv^—Sr1—O1	149.72 (7)	Sr1—O2w—H2w1	104.7
O5^iii^—Sr1—O1	135.70 (8)	Sr1^viii^—O2w—H2w1	104.7
O3^ii^—Sr1—O1	71.79 (7)	Sr1—O2w—H2w2	104.7
O2^i^—Sr1—O1w	145.90 (10)	Sr1^viii^—O2w—H2w2	104.7
O6^iv^—Sr1—O1w	82.82 (8)	H2w1—O2w—H2w2	105.7
O5^iii^—Sr1—O1w	99.51 (9)	C2—C1—C6	119.8 (3)
O3^ii^—Sr1—O1w	135.57 (9)	C2—C1—H1	120.1
O1—Sr1—O1w	81.56 (8)	C6—C1—H1	120.1
O2^i^—Sr1—O2w	74.84 (7)	C1—C2—C3	121.5 (3)
O6^iv^—Sr1—O2w	75.06 (7)	C1—C2—S1	120.3 (2)
O5^iii^—Sr1—O2w	147.45 (7)	C3—C2—S1	118.1 (2)
O3^ii^—Sr1—O2w	130.00 (7)	C4—C3—C2	120.1 (3)
O1—Sr1—O2w	75.62 (7)	C4—C3—H3	120.0
O1w—Sr1—O2w	72.99 (10)	C2—C3—H3	120.0
O2^i^—Sr1—O2w^v^	138.66 (7)	C3—C4—C5	120.3 (3)
O6^iv^—Sr1—O2w^v^	134.37 (7)	C3—C4—H4	119.8
O5^iii^—Sr1—O2w^v^	73.79 (7)	C5—C4—H4	119.8
O3^ii^—Sr1—O2w^v^	63.25 (7)	C4—C5—C6	119.4 (3)
O1—Sr1—O2w^v^	64.07 (7)	C4—C5—C10	121.3 (3)
O1w—Sr1—O2w^v^	73.33 (9)	C6—C5—C10	119.2 (3)
O2w—Sr1—O2w^v^	130.23 (8)	C1—C6—C7	122.6 (3)
O2—S1—O3	113.41 (13)	C1—C6—C5	118.8 (3)
O2—S1—O1	112.68 (14)	C7—C6—C5	118.6 (3)
O3—S1—O1	110.90 (13)	C8—C7—C6	120.6 (3)
O2—S1—C2	107.06 (13)	C8—C7—H7	119.7
O3—S1—C2	106.29 (14)	C6—C7—H7	119.7
O1—S1—C2	105.92 (13)	C7—C8—C9	121.2 (3)
O6—S2—O5	113.63 (13)	C7—C8—S2	120.7 (2)
O6—S2—O4	112.04 (16)	C9—C8—S2	118.0 (2)
O5—S2—O4	111.67 (15)	C10—C9—C8	119.4 (3)
O6—S2—C8	107.02 (14)	C10—C9—H9	120.3
O5—S2—C8	104.65 (14)	C8—C9—H9	120.3
O4—S2—C8	107.23 (14)	C9—C10—C5	120.8 (3)
S1—O1—Sr1	123.13 (12)	C9—C10—H10	119.6
S1—O2—Sr1^ii^	149.15 (13)	C5—C10—H10	119.6
			
O2—S1—O1—Sr1	−99.69 (16)	O2—S1—C2—C1	1.7 (3)
O3—S1—O1—Sr1	28.67 (19)	O3—S1—C2—C1	−119.8 (3)
C2—S1—O1—Sr1	143.58 (13)	O1—S1—C2—C1	122.2 (3)
O2^i^—Sr1—O1—S1	−14.73 (16)	Sr1—S1—C2—C1	151.7 (2)
O6^iv^—Sr1—O1—S1	−100.53 (18)	O2—S1—C2—C3	179.3 (2)
O5^iii^—Sr1—O1—S1	104.78 (16)	O3—S1—C2—C3	57.8 (2)
O3^ii^—Sr1—O1—S1	55.81 (15)	O1—S1—C2—C3	−60.2 (3)
O1w—Sr1—O1—S1	−160.21 (17)	Sr1—S1—C2—C3	−30.7 (3)
O2w—Sr1—O1—S1	−85.71 (15)	C1—C2—C3—C4	−1.4 (4)
O2w^v^—Sr1—O1—S1	124.29 (16)	S1—C2—C3—C4	−179.0 (2)
O3—S1—O2—Sr1^ii^	−22.5 (3)	C2—C3—C4—C5	0.6 (4)
O1—S1—O2—Sr1^ii^	104.6 (3)	C3—C4—C5—C6	0.9 (4)
C2—S1—O2—Sr1^ii^	−139.4 (2)	C3—C4—C5—C10	−178.0 (3)
Sr1—S1—O2—Sr1^ii^	64.1 (3)	C2—C1—C6—C7	−179.7 (3)
O2—S1—O3—Sr1^i^	−62.7 (2)	C2—C1—C6—C5	0.7 (4)
O1—S1—O3—Sr1^i^	169.39 (18)	C4—C5—C6—C1	−1.5 (4)
C2—S1—O3—Sr1^i^	54.7 (2)	C10—C5—C6—C1	177.4 (3)
Sr1—S1—O3—Sr1^i^	−173.4 (2)	C4—C5—C6—C7	178.9 (3)
O6—S2—O5—Sr1^vi^	72.0 (3)	C10—C5—C6—C7	−2.2 (4)
O4—S2—O5—Sr1^vi^	−160.0 (2)	C1—C6—C7—C8	−178.4 (3)
C8—S2—O5—Sr1^vi^	−44.4 (3)	C5—C6—C7—C8	1.1 (5)
O5—S2—O6—Sr1^vii^	0.2 (3)	C6—C7—C8—C9	1.4 (5)
O4—S2—O6—Sr1^vii^	−127.6 (2)	C6—C7—C8—S2	−174.7 (2)
C8—S2—O6—Sr1^vii^	115.2 (3)	O6—S2—C8—C7	−13.3 (3)
O2^i^—Sr1—O2w—Sr1^viii^	37.85 (9)	O5—S2—C8—C7	107.5 (3)
O6^iv^—Sr1—O2w—Sr1^viii^	−43.75 (10)	O4—S2—C8—C7	−133.7 (3)
O5^iii^—Sr1—O2w—Sr1^viii^	−49.76 (17)	O6—S2—C8—C9	170.5 (2)
O3^ii^—Sr1—O2w—Sr1^viii^	93.43 (12)	O5—S2—C8—C9	−68.6 (3)
O1—Sr1—O2w—Sr1^viii^	143.91 (11)	O4—S2—C8—C9	50.1 (3)
O1w—Sr1—O2w—Sr1^viii^	−130.67 (11)	C7—C8—C9—C10	−2.7 (5)
O2w^v^—Sr1—O2w—Sr1^viii^	180.0	S2—C8—C9—C10	173.4 (2)
S1—Sr1—O2w—Sr1^viii^	123.51 (9)	C8—C9—C10—C5	1.6 (5)
C6—C1—C2—C3	0.7 (4)	C4—C5—C10—C9	179.7 (3)
C6—C1—C2—S1	178.3 (2)	C6—C5—C10—C9	0.8 (4)

Symmetry codes: (i) −*x*, −*y*+1, *z*−1/2; (ii) −*x*, −*y*+1, *z*+1/2; (iii) −*x*+1/2, *y*+1/2, *z*+1/2; (iv) −*x*+1/2, *y*+1/2, *z*−1/2; (v) *x*, *y*, *z*+1; (vi) −*x*+1/2, *y*−1/2, *z*−1/2; (vii) −*x*+1/2, *y*−1/2, *z*+1/2; (viii) *x*, *y*, *z*−1.

### Hydrogen-bond geometry (Å, °)

**Table:**

*D*—H···*A*	*D*—H	H···*A*	*D*···*A*	*D*—H···*A*
O1w—H1w1···O4^ix^	0.84	2.29	3.066 (4)	154
O1w—H1w2···O4^x^	0.84	2.27	2.904 (4)	132
O2w—H2w2···O4^x^	0.84	2.03	2.856 (3)	167

Symmetry codes: (ix) −*x*+1, −*y*+1, *z*+1/2; (x) −*x*+1, −*y*+1, *z*−1/2.

## References

[bb1] Barbour, L. J. (2001). *J. Supramol. Chem.* **1**, 189–191.

[bb2] Cai, J.-W. (2004). *Coord. Chem. Rev.* **248**, 1061–1083.

[bb3] Cai, J., Chen, C.-H., Liao, C.-Z., Feng, X.-L. & Chen, X.-M. (2001). *Acta Cryst.* B**57**, 520–530. 10.1107/s010876810100862x 11468379

[bb4] Flack, H. D. (1983). *Acta Cryst.* A**39**, 876–881.

[bb5] Higashi, T. (1995). *ABSCOR* Rigaku Corporation, Tokyo, Japan.

[bb6] Rigaku (1998). *RAPID-AUTO* Rigaku Corporation, Tokyo, Japan.

[bb7] Rigaku/MSC (2002). *CrystalClear* Rigaku/MSC Inc., The Woodlands, Texas, USA.

[bb8] Sheldrick, G. M. (2008). *Acta Cryst.* A**64**, 112–122. 10.1107/S0108767307043930 18156677

[bb9] Westrip, S. P. (2010). *J. Appl. Cryst.* **43**, 920–925.

